# Variation of physical durability between LLIN products and net use environments: summary of findings from four African countries

**DOI:** 10.1186/s12936-020-03549-2

**Published:** 2021-01-07

**Authors:** Albert Kilian, Emmanuel Obi, Paul Mansiangi, Ana Paula Abílio, Khamis Ameir Haji, Sean Blaufuss, Bolanle Olapeju, Stella Babalola, Hannah Koenker

**Affiliations:** 1 PMI VectorWorks Project, Tropical Health LLP, Montagut, Spain; 2 PMI VectorWorks Project, Tropical Health LLP, Abuja, Nigeria; 3 Ecole de Santé Publique, Université de Kinshasa, Kinshasa, Democratic Republic of Congo; 4 Instituto Nacional de Saúde, Maputo, Mozambique; 5 Zanzibar Malaria Elimination Programme, Stone Town, Zanzibar, Tanzania; 6 PMI VectorWorks Project, JHU Center for Communication Programs, Baltimore, MD USA

**Keywords:** LLIN physical durability, Textile resistance to damage

## Abstract

**Background:**

Physical durability of long-lasting-insecticidal nets (LLIN) is an important aspect of the effectiveness of LLIN as a malaria prevention tool, but there is limited data on performance across locations and products. This secondary analysis of data from the VectorWorks project from 10 sites in four African countries involving six LLIN brands provides such data.

**Methods:**

A total of 4672 campaign nets from 1976 households were recruited into prospective cohort studies 2–6 months after distribution through campaigns and followed for 3 years in Mozambique, Nigeria, DRC and Zanzibar, Tanzania. LLIN products included two 100 denier polyester LLIN (DawaPlus^®^ 2.0, PermaNet^®^ 2.0) distributed in five sites and four 150 denier polyethylene LLIN (Royal Sentry^®^, MAGNet^®^, DuraNet©, Olyset™ Net) distributed in five sites. Primary outcome was LLIN survival in serviceable condition and median survival in years. Net use environment and net care variables were collected during four household surveys. Determinants of physical durability were explored by survival analysis and Cox regression models with risk of failure starting with the first hanging of the net.

**Results:**

Definite outcomes for physical durability were obtained for 75% of study nets. After 31 to 37 months survival in serviceable condition varied between sites by 63 percentage-points, from 17 to 80%. Median survival varied by 3.7 years, from 1.6 to 5.3 years. Similar magnitude of variation was seen for polyethylene and polyester LLIN and for the same brand. Cox regression showed increasing net care attitude in combination with exposure to net related messages to be the strongest explanatory variable of survival. However, differences between countries also remained significant. In contrast, no difference was seen for LLIN material types.

**Conclusions:**

Variation in net use environment and net care is the main reason for differences in the physical durability of LLIN products in different locations. While some of these factors have been identified to work across countries, other factors remain poorly defined and further investigation is needed in this area. Grouping LLIN brands by similar textile characteristics, such as material or yarn strength, is insufficient to distinguish LLIN product performance suggesting a more differentiated, composite metric is needed.

## Background

Early field testing of long-lasting insecticidal nets (LLIN) had focussed mainly on the insecticidal aspects following the early guidance on phase III assessments of the World Health Organization’s (WHO) Pesticide Evaluation Scheme (WHOPES) [1]. For a LLIN product to receive WHOPES recommendation for public health use it had to demonstrate that after 3 years in the field at least 80% of the LLIN still showed optimal insecticidal effectiveness based on bio-assay tests in the laboratory (cone or tunnel test). Aspects of physical durability were not part of these early assessments even though increasing damage over time of use in mosquito nets had been highlighted as an issue in six studies published between 1982 and 2004 [2–7]. The first study to report on the physical integrity of an LLIN was published 2004 when Spencer et al. [8] presented data on PermaNet^®^ 1.0 distributed in a camp for internally displaced people in Bundibugyo district, Uganda. Damage in this study was described in two categories, any holes below or above 40 cm^2^ in size. The first studies using a single composite metric of damage across each LLIN were presented for Olyset™ Net from the Lao Republic in 2007 using the total surface area of holes per net [9] and for PermaNet^®^ 1.0 and 2.0 in 2008 from Uganda using a simple hole index based on the count of three hole sizes [10]. These early studies did not yet include standardized measurement of loss of nets due to decay (attrition) and were only describing one LLIN brand in each location. In addition to Olyset™ Net and PermaNet^®^ 2.0 the polyester-based Interceptor^®^ LLIN was studied in Uganda [11] and India [12].

The first paper presenting data comparing physical durability of more than one LLIN brand in the same location was published in 2012. Using an early version of a “proportionate hole index” as a composite measure of net damage the 150 denier polyethylene-based Olyset™ Net was compared with two polyester-based 75 denier LLIN, PermaNet^®^ 2.0 and Interceptor^®^ in the settlements of internally displaced people in Chad [13]. After 14 months of use 39% of the polyester-based LLIN were considered no longer serviceable compared to only 8% of the polyethylene-based LLIN with a thicker yarn. However, the study only looked at the surviving nets and did not capture losses due to nets thrown away because of damage (attrition) which may have distorted the results if attrition was higher in the Olyset™ Net group. In 2013 WHO published a new recommended methodology for the monitoring of the physical durability of LLIN that combined attrition due to wear and tear and integrity of LLIN still found in the sampled households into a “functional survival in serviceable condition” [14]. Using this new approach van Roey and colleagues compared the 120 denier polyethylene-based Netprotect^®^ LLIN in the same district in Cambodia to the 100 denier polyester-based PermaNet^®^ 2.0 and found no difference in functional survival after 3 years of follow-up [15]. Other brand comparisons of physical durability that were done in the same locations compared Olyset™ Net against PermaNet^®^ 2.0 and no differences were found between them in Rwanda [16], India [17], Uganda [18] or Zambia [19]. In Nampula province in Mozambique Olyset™ Net had a higher mean number of holes per net after 2 years, but this study did not include attrition [20]. In contrast, after 3 years follow-up of the 100 denier polyester-based DawaPlus^®^ 2.0 LLIN in three different locations in Nigeria a significant variation in median survival of over 1 year was found [21] suggesting that net use environment may be the driving force of differences in physical durability of LLIN.

The first study comparing physical durability of more than two LLIN brands in a three-year prospective cohort using the new methodology and controlling for location by randomly allocating nets to households in ten districts in Tanzania included two polyethylene-based LLINs, Olyset™ Net and Netprotect^®^, and one polyester-based LLIN, PermaNet^®^ 2.0 [22]. While estimated median survival in serviceable condition was the same for PermaNet^®^ 2.0 and Netprotect^®^ with 2.6 years, there was strong statistical evidence that median survival for Olyset™ Net was lower with 2.0 years (p < 0.001). A study in Benin randomly distributed seven LLIN brands in one sub-district, four polyester-based, one polyester LLIN with enforced border and polyethylene roof (PermaNet 3.0) and two polyethylene-based LLIN and follow-up data up to 12 months did not suggest any differences by brand [23]. Finally, a recent secondary analysis and modelling of data from 3-year durability monitoring from seven countries involving between one and seven LLIN brands per country and using vectorial capacity as the primary outcome found more variability in decline of protection over time by country than by LLIN brand [24].

The VectorWorks project, funded by the U.S. President’s Malaria Initiative (PMI) undertook LLIN durability monitoring between 2014 and 2019 involving four African countries and five LLIN brands (Table 1) [25–28]. The secondary analysis of this data across locations and LLIN brands not only presents a further opportunity to investigate variations of physical durability for the same or similar (same specifications but different manufacturer) LLIN between different net use environments, but also allows adjustment of comparison between LLIN types for the key elements of net care behaviours as these were comprehensively collected in a standardized fashion across all project sites.

**Table 1:** Countries, locations, and LLIN brands of durability monitoring

Country	Province (State)	District (Local Government Area, Health Zone,)	LLIN brand
Mozambique MOZ	Inhambane	Jangamo	Royal Sentry^®^
Mozambique MOZ	Nampula	Angoche	Royal Sentry^®^
Mozambique MOZ	Tete	Changara	MAGNet^®^
Nigeria NGA	Ebonyi	Ishielu	DawaPlus 2.0^®^
Nigeria NGA	Oyo	Akinyele	DawaPlus 2.0^®^
Nigeria NGA	Zamfara	Bakura	DawaPlus 2.0^®^
Democratic Republic Congo DRC	Mongala	Binga	DawaPlus 2.0^®^
Democratic Republic Congo DRC	Ubangi Sud	Ndege	DuraNet©
Zanzibar (Tanzania) ZNZ	Pemba	Wete	Olyset™ Net
Zanzibar (Tanzania) ZNZ	Unguja	North B	PermaNet 2.0^®^

## Methods

### Study sites

Data from 10 sites of durability monitoring activities in four countries involving six LLIN brands were included in the analysis and details of locations and LLIN brands studied are shown in Table 1. There were two distinct country scenarios. In Mozambique and Nigeria the same or similar LLIN brands were tested in what was expected to be different net use environments, while in the Democratic Republic of Congo (DRC) and Zanzibar different LLIN brands were monitored in similar locations. The selection of the scenario was based on the information needs of the country’s malaria programme.

### Primary data collections

The study design was the same in all countries and followed a standardized protocol recommended by the PMI [29] and in line with WHO recommendations [14]. Details of the methodology and tools have been presented previously [25–28]. In short, a representative sample of LLINs distributed through a mass distribution campaign organized by the respective malaria programmes were recruited into a prospective cohort study 1 to 6 months after distribution. Sample size target was 345 cohort nets per site sampled from 15 clusters per site and 10 households per cluster in Nigeria, Zanzibar and DRC. In Mozambique sample size was higher with 782 cohort nets targeted per site from 17 clusters. These differences were due to varying assumptions for precision of estimates. Clusters were selected with probability proportionate to size and households were selected by simple random sampling from lists prepared at the day of the survey. Follow-up surveys were conducted 12, 24 and 36 months after distribution. At each time point presence or loss of the nets were recorded (attrition) and an assessment of the physical integrity of the remaining cohort nets was carried out. During hole count fully repaired holes were only recorded as repair while partial repairs were counted as the remaining hole and a repair. Data collections took place between November 2015 and April 2019. Follow-up in Oyo State, Nigeria only was for 24 months due to a delay in the LLIN mass campaign and the end of the VectorWorks project. All other sites completed the 36 months follow-up survey. Data was collected electronically using tablets and the Open Data Kit (ODK) software. After data cleaning and consistency checks, data were transferred to the Stata statistical package (Stata version 14.2, College Station, Texas, USA) for processing and analysis.

Physical integrity was measured by the proportionate Hole Index (pHI) as recommended by WHO [30] and then categorized based on the pHI value as still serviceable (pHI ≤ 642) or torn (pHI > 642) [31]. Primary outcome of the physical durability assessment was the survival in serviceable condition which incorporates attrition due to discarding of nets (destroyed, thrown away or used for other purposes) and surviving nets no longer serviceable. Nets that were given to others to use or for which outcome was unknown were excluded from the uni- and bivariate analyses and censored in the survival analysis [30].

Median net survival was estimated defined as the time in years until 50% of the originally distributed LLIN were no longer serviceable. It was calculated from at the last two time points provided both were below 85% using the following formula:

$${\text{tm}} = {\text{t}}1 + \frac{{\left( {{\text{t}}2 - {\text{t}}1} \right) *\left( {{\text{p}}1 - 50} \right)}}{{\left( {{\text{p}}1 - {\text{p}}2} \right)}}$$

where tm is the median survival time, t1 and t2 the first and second time points in years and p1 and p2 the proportion surviving to first and second time point respectively in per cent. Confidence intervals for this estimate were calculated by projecting the 95% CI from the survival estimates in the same way as described above.

In addition, information on socio-demographic characteristics, ownership of other mosquito nets, net use environment, net handling, and net care and repair behaviour was collected through household-level questionnaires. Specifically, a household net care attitude variable was developed based on a Likert scale comprising six questions with a four-value response, omitting the neutral option. Based on this variable households were categorized as never, sometimes or always showing very positive net care attitude (score ≥ 1.0 from a range − 2.0 to 2.0) across the up to four surveys each household participated in [26]. Similarly, a variable of household exposure to social and behaviour change (SBC) messages regarding LLIN was created with categories of being exposed “never”, “at least once” or “twice or more” during the course of the study.

### Secondary data analysis

For this secondary analysis four types of data sets were used from each country, the household and cohort LLIN master lists and the household and cohort net result file including all observations across all four surveys per site. These data sets were then merged and unique identifiers created for each cohort net and household within each site. Based on the findings of the separate country data analyses on the relationship between SBC message exposure and net care attitude a new variable was created for the secondary analysis that combined the two variables into four groups as follows: (i) never positive net care attitude and never SBC exposed; (ii) never positive net care attitude and one or more SBC exposures; (iii) at least one positive care attitude combined with any number of SBC exposures; (iv) at least twice positive net care attitude and at least twice exposed to SBC messages.

### Statistical analysis

Data was set up for survival analysis as a duration format data set where each time interval for a net was a separate observation. Survival analysis was done using a per-protocol approach, i.e. risk of failure was considered to start only on the first observation where the net was found hanging, i.e. excluding any net that was never hung as well as the time period to first hanging. Failure was defined as a net being reported lost to wear and tear or torn based on physical assessment (pHI). The time of failure was directly calculated from the report of time of loss by the respondent or taken as the mid-point between the last two surveys if time of loss was unknown. In addition, at each time point the proportion surviving in serviceable condition was plotted against the hypothetical survival curves with defined median survival as previously described [26].

For continuous variables, arithmetic means were used to describe the central tendency and the t-test for comparison of groups for normally distributed data. Otherwise, median and Kruskal–Wallis test were used. Proportions were compared by contingency tables and the Chi-squared test used to test for differences in proportions. For calculation of confidence intervals around estimates, the intra- and between-cluster correlation was taken into account using the *svy* command in Stata.

Determinants of survival in serviceable condition after the net was first hung were explored using Cox proportionate hazard models. Factors were tested first in individual models which were then used to construct the final multivariate models. Final model fit was tested using a linktest and Schoenfeld residuals and log–log plots were used to check the proportionate hazard assumption.

## Results

The four-country sample included a cohort of 4672 campaign nets from 1976 households. At the end of follow-up, a definite outcome with respect to physical durability could be obtained for 3519 nets or 75%. As shown in Table 2 this proportion varied significantly between sites with the highest rate found in Zamfara (92%), Nigeria, and the lowest in Tete (45%), Mozambique. For 4/10 sites the proportion of cohort nets with definite outcomes was above 85% and for 8/10 over 70%. The most common reasons for loss to follow-up was households moving away or not being available at the time of the surveys and respondents being unable to state what had happened to a cohort net no longer present in the household.

**Table 2:** LLIN sampled into cohorts, definite outcomes and cohort nets ever found hanging

Site	Number of cohort nets recruited	Proportion of cohort nets with definite outcome (95% CI)	Proportion of cohort nets ever found hanging (95% CI)
MOZ Inhambane	726	79.8% (74.9–83.9)	65.7% (61.4–69.8)
MOZ Nampula	661	72.0% (64.4–78.5)	70.2% (63.2–76.4)
MOZ Tete	601	44.9% (34.1–56.3)	75.0% (65.3–82.7)
NGA Ebonyi	367	88.0% (78.7–93.3)	85.3% (79.9–89.5)
NGA Oyo*	372	74.7% (66.6–81.4)	55.1% (48.3–61.7)
NGA Zamfara	357	91.6% (85.2–95.4)	99.4% (96.1–99.9)
DRC Mongala	377	74.3% (63.4–82.8)	54.6% (45.6–63.4)
DRC Ubangi Sud	377	67.4% (58.3–75.2)	82.2% (74.5–88.0)
ZNZ Pemba	452	89.4% (83.5–93.4)	76.3% (67.7–83.2)
ZNZ Unguja	382	85.9% (77.1–91.7)	77.5% (69.8–83.7)

* Only 24-months follow-up

The Oyo site in Nigeria was the only one with a peri-urban characteristic due to its proximity to the city of Ibadan, all others were rural and agricultural communities. House construction was very simple and fuel for cooking was predominantly firewood. Access to latrines was over 80% except in Oyo (32%) and Ebonyi (66%) in Nigeria, and Tete (52%) and Nampula (64%) in Mozambique. Similarly, over 80% of households had access to safe water except in Mongala (18%) and Sud Ubangi (0%) in DRC and Ebonyi (13%), Nigeria where people obtained drinking water from rivers and creeks. The household asset that varied most between sites was mobile phone ownership which was 79–82% in both sites in Zanzibar and Inhambane, Mozambique, 48–69% in Nigeria sites and Nampula, Mozambique and 10-35% in DRC and Tete, Mozambique. In none of the sites was there any evidence of significant changes in socio-economic status of households during the study period.

Most LLINs received from the campaign were not immediately used. Initial hanging and utilization of campaign nets at the baseline survey 2–6 months after distribution was below 50% in 8/10 sites ranging from 13 to 42%. Only in Sud-Ubangi (54%), DRC and Zamfara (77%), Nigeria was it higher. As shown in Table 2, the proportion of nets ever found hanging during the study period varied significantly between sites ranging from 99% in Zamfara, Nigeria to 55% in Mongala, DRC and Oyo, Nigeria. In only slightly more than half of the sites (6/10) did “ever found hanging” exceed 75%. The two main factors that influenced hanging and use of the campaign nets were the number of nets from other sources owned by households at a given time and whether or not the household had been oversupplied during the campaign distribution, i.e. received more than one LLIN for every two members.

**Fig. 1:**
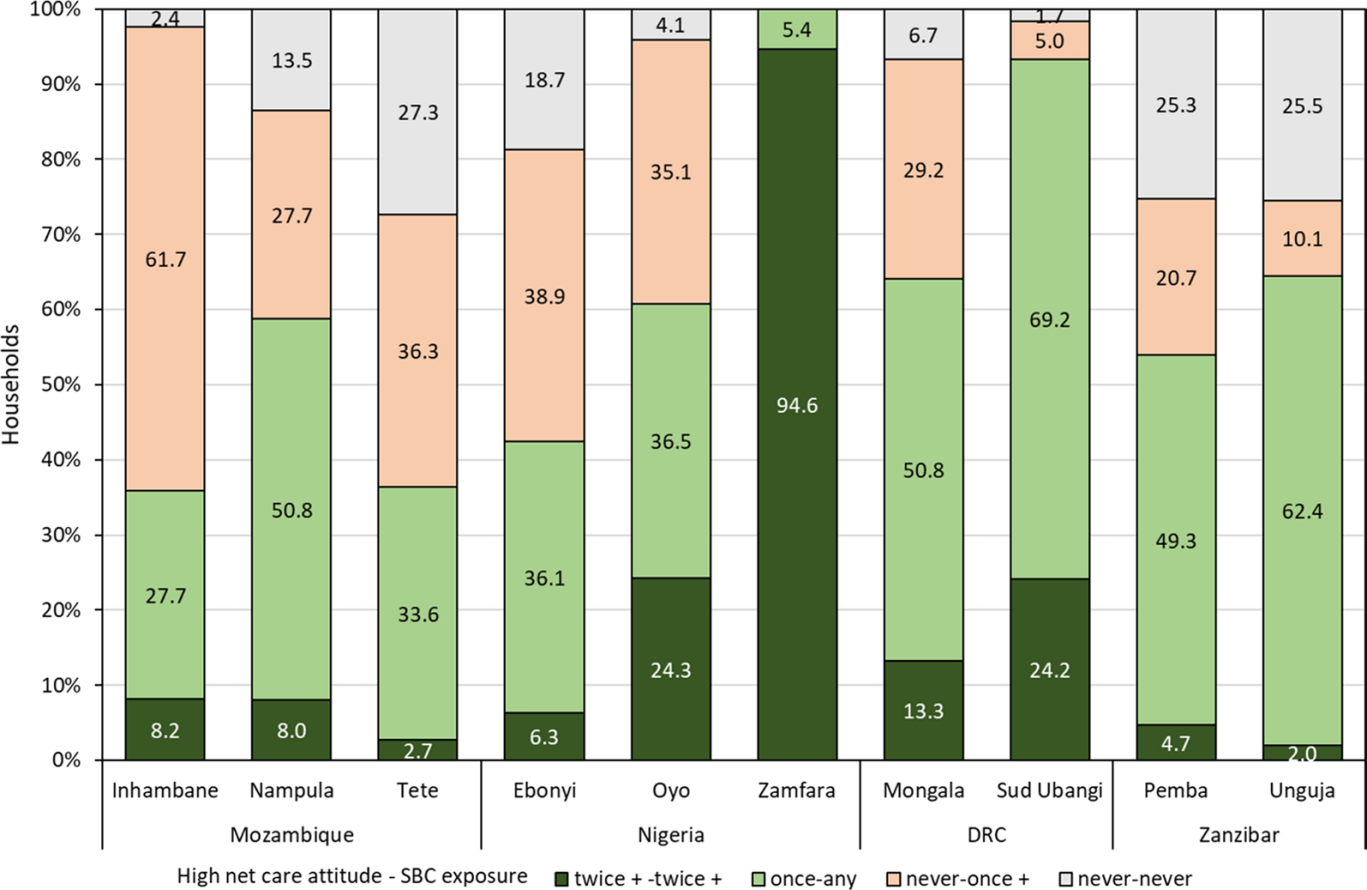
Observations over time of households with high positive net care attitude (score > 1.0) and exposure to SBC messages. Never–never = never high net care attitude, never exposed to SBC messages; never–once+ = never high net care attitude and SBC exposure at least once; once–any = high net care attitude at once and any SBC exposure (none or any); twice+-twice+ = high net care attitude twice or more, SBC exposure twice or more

Overall, 71% of households participated in all possible household surveys (country range 62 to 84%) and 90% (range 87 to 96%) attended three or more surveys. The combined net care attitude and SBC exposure variable showed clear differences between countries and sites (Fig. 1). Zamfara, Nigeria performed best with 95% of households showing consistently high net care attitude and high SBC exposure. Oyo, Nigeria and the DRC sites also performed well but within DRC Sud Ubangi showed higher attitude and SBC exposure than Mongala (p < 0.001). In Mozambique and Ebonyi, Nigeria there was considerable exposure to SBC, but this did not always translate into a positive net care attitude as between 28 and 62% of households had some level of SBC exposure, but never showed a positive net care attitude. Details of trends in SBC exposure and the mix of SBC channels over time have been presented in detail in the country-specific analyses [25–28].

The development of LLIN survival in serviceable condition over time is shown in Table 3. At baseline failure was 1% or less, but already at the 12-months follow-up survey survival was lower than the 92% expected under a 3-year median survival assumption in both DRC sites (70 and 89%) as well as for Pemba (86%), Zanzibar. At the end of follow-up after 31–37 months, survival ranged from 17% in Mongala, DRC, to 80% in Zamfara, Nigeria, a difference of 63 percentage points. The median value at the 36–months time-point was 51% with an inter-quartile range of 18 percentage points (37–55%).

**Table 3:** Survival in serviceable condition at follow-up intervals

Site	Baseline (95% CI)	12 months (95% CI)	24 months (95% CI)	36 months (95% CI)
MOZ Inhambane	99.7% (98.1–99.9)	98.0% (96.0–99.0)	85.3% (78.9–90.0)	57.3% (50.2–64.1)
MOZ Nampula	100% (–.–)	93.7% (90.6–95.8)	73.2% (62.7–81.7)	32.5% (23.5–43.1)
MOZ Tete	98.7% (96.3–99.5)	95.8% (90.7–98.1)	74.2% (64.2–82.1)	43.3% (27.2–61.1)
NGA Ebonyi	99.7% (98.0–100)	96.0% (92.5–97.9)	76.3% (67.9–83.1)	54.8% (41.4–67.6)
NGA Oyo	100% (–.–)	92.0% (86.0–95.6)	74.6% (60.2–85.1)	n.a.
NGA Zamfara	99.3% (97.9–99.8)	97.7% (95.7–98.8)	91.8% (84.1–95.9)	80.4% (72.7–85.9)
DRC Mongala	98.9% (96.7–99.7)	69.6% (59.5–78.1)	33.2% (23.5–44.4)	17.4% (10.7–26.9)
DRC Ubangi Sud	100% (–.–)	88.7% (84.8–91.7)	56.2% (45.7–66.1)	36.7% (29.4–44.7)
ZNZ Pemba	99.1% (97.8–99.6)	86.1% (78.7–91.2)	67.0% (60.6–72.6)	51.0% (44.5–57.4)
ZNZ Unguja	99.2% (96.7–99.8)	93.9% (89.6–96.4)	75.8% (67.1–82.8)	55.2% (46.2–63.9)

**Fig. 2:**
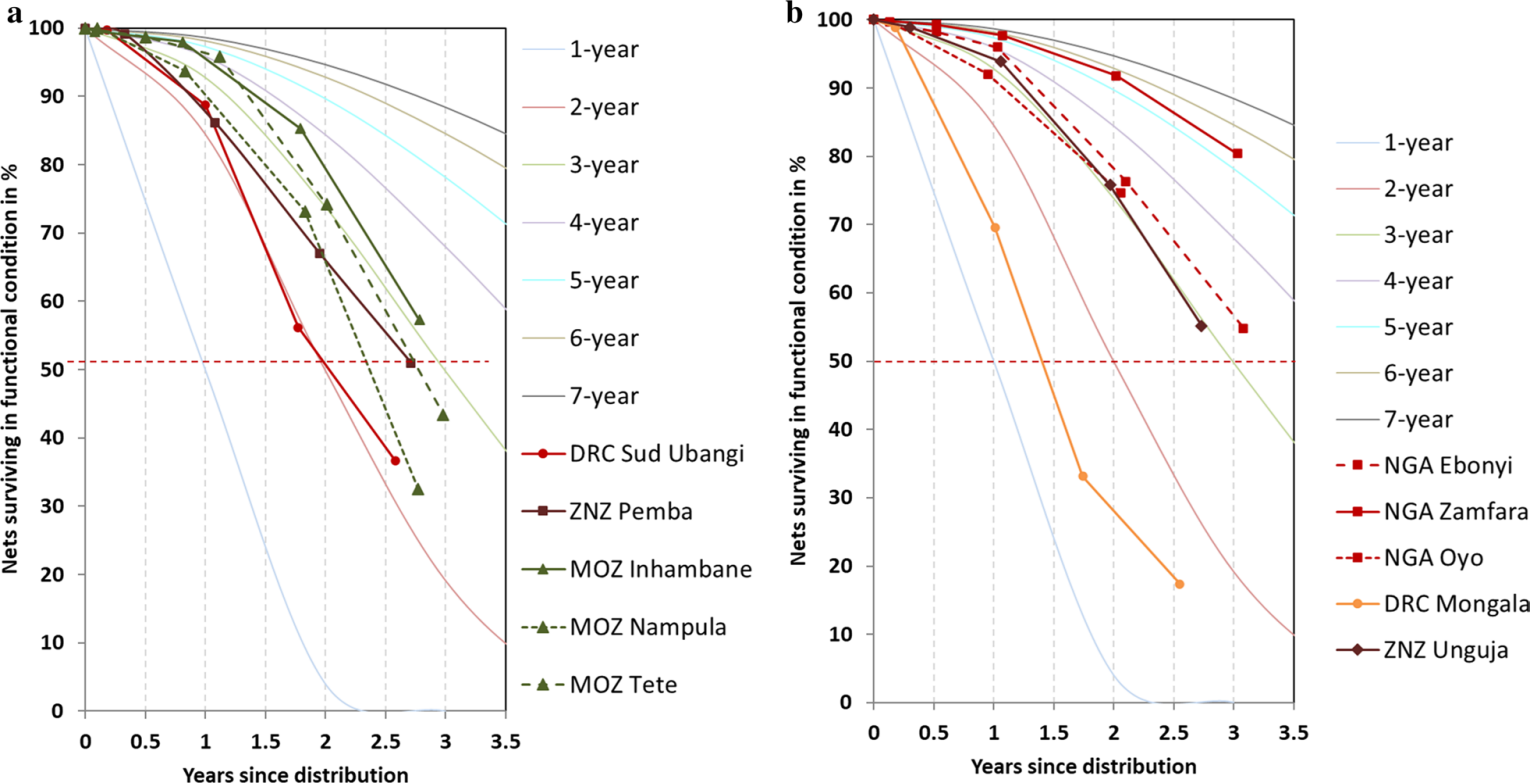
Variation in survival in serviceable condition by site and LLIN type. A = polyethylene 150 denier LLIN; B = polyester 100 denier LLIN, reference curves refer to hypothetical survival curves with defined median survival

When plotted against the hypothetical survival curves with defined median survival (Fig. 2) most curves generally followed the s-shaped form of the hypothesized curves with a tendency of slightly higher survival estimates at 12 months compared to 24 and 36-months follow-up. The one exception was the Olyset™ Net in Pemba, Zanzibar (Fig. 2a) which started out near the 2-year median survival line but then gradually improved and ended up closer to the 3-year median survival line at 36 months. The country analysis suggests that this was at least in part due to an improvement of net care behaviour during the course of the study [27]. In Fig. 2 data on survival in serviceable condition is grouped by textile characteristics and shows that the same or very similar LLIN brands had a huge variation in physical durability performance. This is further confirmed by the estimated median survival in serviceable condition and their confidence intervals shown in Table 4. For the DawaPlus^®^ 2.0 LLIN median survival time varied between 1.6 and 5.3 years between sites and for the Royal Sentry LLIN between 2.4 and 3.0 years. If all polyethylene-based, 150 denier LLIN brands were considered between-site variation ranged from 2.2 to 3.0 years. The median of the median survival estimates across the 10 sites was 2.9 years with an inter-quartile range of 0.8 years (2.4 to 3.2).

**Table 4:** Estimated median survival in serviceable condition by LLIN brand

LLIN Brand	Material and denier	Site	Median survival in years	95% CI of median survival
DawaPlus 2.0^®^	PET–100	NGA Ebonyi	3.3	2.8–4.2
DawaPlus 2.0^®^	PET–100	NGA Oyo	3.2	2.1–5.3
DawaPlus 2.0^®^	PET–100	NGA Zamfara	5.3	4.6–6.4
DawaPlus 2.0^®^	PET–100	DRC Mongala	1.6	1.3–1.9
PermaNet 2.0^®^	PET–100	ZNZ Unguja	2.9	2.5–3.3
Royal Sentry^®^	PE–150	MOZ Inhambane	3.0	2.8–3.3
Royal Sentry^®^	PE–150	MOZ Nampula	2.4	2.1–2.6
MAGNet^®^	PE–150	MOZ Tete	2.8	2.4–3.5
DuraNet©	PE–150	DRC Ubangi Sud	2.2	2.0–2.4
Olyset™ Net	PE–150	ZNZ Pemba	2.7	2.5–3.0

PE: polyester; PET: polyethylene

The variation between sites of the proportion of studied campaign nets used immediately after distribution or ever found hanging (Table 2) can be expected to influence the median survival estimates upwards, but it is noteworthy, that the site with the highest survival (Zamfara, Nigeria) also had the highest proportion of nets ever hung as well as the highest proportion of households with a very high net care attitude score. In order to exclude the impact of delayed use of nets during exploration of determinants of physical durability across countries the per-protocol approach was used in which the risk of failure to survive only started with the first observed hanging of the net. Results for the final cox regression model are shown in Table 5. As was the case in the individual country analyses [25–28], a strong household net care attitude was the strongest determinant of survival and was enhanced by exposure to SBC messages. Adjusting for other factors, LLIN from households with some SBC exposure but which never recorded a high net care attitude had a 30% better chance of survival (adjusted Hazard Ratio (aHR) 0.70) than LLIN from households which never reported SBC exposure and never recorded a high net care attitude. The survival advantage doubled to 65% (aHR 0.35) if the household was recorded with a high net care attitude at least twice during the study and also had been exposed the SBC messages at least twice. Plotting the adjusted survival curves for each determinant in Table 5 shows that LLINs from households in the twice–twice category gain approximately one full year of median survival compared to the never–never category of net care attitude and SBC exposure (Additional file 1: A). Other determinants of physical durability that improved survival were never cooking in the room where nets were hanging, nets being used by adults rather than only by children, nets from households in the highest wealth group, and nets in female headed households. A negative effect was noted for never folding the net up during the day, i.e. letting it hang loose over the sleeping place. However, none of these factors had as strong of an impact on survival as net care attitude and SBC exposure (see also Additional file 1: D–H).

**Table 5:** Determinants of physical survival of LLIN in a per-protocol Cox regression model

Variable	Adjusted hazard ratio (aHR)	95% CI	p-value
N = 5126 obs/2900 nets			
High net care attitude score and SBC exposure			
Attitude never–SBC never	1.00		
Attitude never–SBC at least once	0.70	0.56–0.89	0.003
Attitude at least once–SBC never or at least once	0.57	0.46–0.71	< 0.0001
Attitude at least twice–SBC at least twice	0.35	0.26–0.46	< 0.0001
Type of LLIN			
Polyester 100D	1.00		
Polyethylene 150D	0.94	0.77–1.16	0.58
Never folding net up during day when hanging	1.41	1.18–1.69	< 0.0001
Never cooking inside the sleeping room	0.86	0.75–0.98	0.03
Dominant net users			
Child only	1.00		
Child with adult	0.83	0.68–1.01	0.07
Adult only	0.70	0.57–0.84	< 0.0001
Wealth tertile			
Lowest	1.00		
Middle	0.92	0.78–1.08	0.29
Highest	0.81	0.69–0.96	0.01
Gender of head of household			
Male	1.00		
Female	0.81	0.65–1.01	0.06
Country			
Mozambique	1.00		
Nigeria	0.65	0.46–0.90	0.009
DRC	1.94	1.59–2.39	< 0.0001
Zanzibar (Tanzania)	1.27	1.00–1.64	0.06

No difference in survival was found between the LLIN types of 150 denier polyethylene-based compared to 100 denier polyester-based LLIN products after adjusting for other determinants even though the polyethylene-based LLIN had a slight advantage (Additional file 1: B). However, the final model still indicated a significant impact of the country variable. Specifically, LLIN survival was better in Nigeria compared to Mozambique, marginally poorer in Zanzibar and significantly poorer in DRC (Additional file 1: C). Variations by site within countries was not tested in this model as it coincided with the brand, but these differences have been described previously in the country analyses [25–28]. Variables tested but with no impact included educational status of head of household, storage of food in sleeping rooms and type of sleeping place.

## Discussion

This secondary analysis of pooled data from standardized LLIN durability monitoring at 10 sites in four African countries allowed a look at the magnitude of variation in physical durability of LLIN beyond each individual country analysis. At the 36-months follow-up, which happened between 31 and 37 months post-distribution, the proportion of any LLIN product surviving in serviceable condition was found to vary by 63 percentage-points from 17 to 80% with an inter-quartile range of 18 percentage-points. Even when results were standardized for time of follow-up by expressing them as median years of survival, i.e. time until 50% of the LLIN had failed to be of service to the user, there was a 3.7 year difference between results from the 10 sites (1.6 to 5.3 years) with an inter-quartile range of 0.8 years.

There are a limited number of studies that used the new standardized methodology and either proportion of LLIN surviving in serviceable condition or a median survival estimate in years for comparison of LLIN in different locations. In Benin, Olyset™ Net was monitored in four districts, two in the North and two in the South of the country and after only 18 months the survival in serviceable condition varied between 49 and 65% (16 percentage-points) equivalent to a median survival of 1.5 to 1.9 years [32]. In a retrospective study design survival in serviceable condition of DawaPlus^®^ 2.0 in three locations in Nigeria varied between 42 and 75% (33 percentage-points) after 3 years corresponding to 3.0 to 4.7 years of median survival [21]. And in a multi-brand, multi-country analysis of durability data from seven countries involving eight LLIN brands survival varied for the same brand between countries from 56 to 98% or by between 10 and 42 percentage-points after 3 years of follow-up [24, Additional file 2, Table S1]. These variations by location are significant and in a similar order of magnitude as observed in this study.

Some of the factors of net use environment and net handling and care that are responsible for the variance in LLIN physical durability and are consistent across the four countries have been identified in this secondary analysis. The one with the strongest explanatory value of these was the household net care attitude in connection with exposure to SBC messages about nets and their use, handling and care. The net care attitude score was based on a Likert scale comprising six questions which was then converted into a grouped variable of the frequency in which a household had presented with a very positive net care attitude in all the survey rounds (maximum four) it participated in. Likert scales have long been used in social sciences to capture respondent’s attitudes [33, 34]. Known limitations of this methodology were minimized by omitting the “neutral” response option to reduce central tendency bias [35] and by reversing some statements to minimize acquiescence bias [36]. While it will most likely be possible to further improve on the capture a positive net care attitude of net users, this is currently the best approach available and has been shown in two independent studies to correlate positively with better physical condition of nets [18, 21]. Household net care attitude was positively related with survival of LLIN in serviceable condition in a dose–response relationship, i.e. the stronger the evidence of a positive net care attitude, the greater was the effect of prolonging net life. This was also linked to the intensity of exposure to SBC messages on nets in a way that suggests that a positive attitude was possible even in the absence of SBC, but conversely exposure to SBC was not always sufficient to trigger a positive attitude. This is consistent with other studies that found SBC programmes to increase awareness and knowledge about net use, handing and care, but did not always found a positive effect on actual net care itself reflecting the complexity of the process of behavioural change [18, 37–39].

Some of variables found to be associated with physical durability were actions or behaviours directly related to protecting the LLIN from damage such as folding the net up to keep it out of harm’s way during the day, avoiding open fire or embers in the sleeping room, or better survival of nets used by adults only that can be expected to be more careful in their net handling than younger children. Other explanatory variables were indirect measures of net use environment and/or behaviours such as wealth or female heads of households which can be understood as proxies for more specific factors not yet sufficiently defined or captured in questionnaires. Individual analysis of country data also had shown some factors such as type of sleeping place to be relevant in some countries, but not in others [25–28] and also not in the pooled data. This strongly suggests that there is a lot of collinearity in the variables currently used to describe net use environment and net handling and care, e.g. a finished bed frame is likely associated with adult net users as well as better off households, so that only the strongest explanatory variables appear consistently as determinants of physical durability while others vary depending on the local constellation. After testing all possible variables in the data set of this study the Cox regression still indicated a significant effect of the country variable which implies that some important aspects were not captured at all. While variables used during the VectorWorks project in a standardized fashion represent some progress, there is definitely room for improvement in development of a comprehensive methodology to capture net use environment and behaviours relevant to physical LLIN durability. This could involve improvement of the questions used for net attitude assessment as well as addition of other behavioural aspects based on further qualitative research.

The data presented in this study suggests that differences in physical durability of LLIN products was driven by the location and not the LLIN brand. This is confirmed by the analysis of a large pooled data set of seven countries and eight LLIN brands followed between 2 and 4 years by Briët et al. [24], that found that for both survival and physical integrity there was significantly more variability of decline of protection over time by country than by LLIN brand. In country by country analysis of the data included in this study DuraNet© was shown to perform significantly better with respect to physical durability than DawaPlus^®^ 2.0 in DRC [28] and PermaNet^®^ 2.0 somewhat better than Olyset™ Net after adjusting for net care attitudes in Zanzibar [27] suggesting that in specific environments differences by textile characteristics of LLIN do exist. This was also found in mainland Tanzania where PermaNet^®^ 2.0 and Netprotect^®^ performed better than Olyset™ Net [22]. However, in Zambia no difference between PermaNet^®^ 2.0 and Olyset™ Net could be found [19] nor was there a difference between PermaNet^®^ 2.0 and Netprotect^®^ in the same use environment in Cambodia [15].

It appears, therefore, that any differences by LLIN product characteristics are more subtle and at times can only be detected after adjustment for remaining differences in net handing and care and where use conditions are particularly difficult such as in DRC. Furthermore, results from the Cox regression in this study show that differences in LLIN product performance cannot be captured by rough categories of textile qualities such as yarn material and mass per length (denier). This suggests that additional elements that may be critical for the development or aggravation of damage have to be considered [40, 41].

## Limitations

Some of the durability risk factors, such as net care attitude as well as some of the outcomes, such as reason for net losses were based on the answers of the household members interviewed and therefore, are prone to recall or social desirability biases. With the prospective design there is also the potential for the Hawthorne effect, whereby being asked about net care and handling four times over the course of 3 years may have contributed to changes in behaviour. The standard durability monitoring approach applied in this study tries to minimize this by conducting only four surveys versus every 6 months as had been done in some of the earlier studies. This study is further limited as the selection of LLIN brands was opportunistic, depending on which brands happened to be distributed in the countries where the project supported the LLIN durability activities resulting in not all brands present in all locations.

## Conclusions

Variation in net use environment and net handling and care behaviour is the main factor to explain differences in the physical durability of different LLIN products in different locations. This emphasizes the need to always consider net use environment and care in the assessment of physical LLIN durability of LLIN brands. It further suggests that SBC interventions to improve net care should be strengthened. While some of these factors such as net care attitude and folding up nets during the day have been identified to work across countries, other factors remain poorly defined and more work is needed in this area.

Grouping LLIN brands by similar textile characteristics, namely 150 denier polyethylene-based versus 100 denier polyester-based LLIN is insufficient to distinguish LLIN product performance after adjustment for other factors suggesting that a more differentiated, composite metric is needed that would describe an LLIN’s ability to resist the stresses of day-to-day use and would be based on the detailed study of the mechanisms involved in hole formation.

## Supplementary information

**Additional file 1: Figure S1**. adjusted survival curves for determinants of physical survival from cox regression. Contains survival graph for each variable in the final Cox regression model.

## Data Availability

The datasets used and/or analysed during the current study are available from the corresponding author on reasonable request.
