# Supplementary material for: Variation of physical durability between LLIN products and net use environments: summary of findings from four African countries
Source: Malar J. 2021 Jan 7;20:26. doi: 10.1186/s12936-020-03549-2 (PMC7791654; doi:10.1186/s12936-020-03549-2)

Additional file 1

Figure: adjusted survival curves for determinants of physical survival from cox regression

Legend **A:** high net care attitude and SBC exposure, blue: never-never, red: never-once+, green once-any, yellow: twice+-twice+; **B**: LLIN type, blue: polyester 100 denier, red: polyethylene150 denier; **C:** country: yellow: DRC, green: Zanzibar, blue Mozambique, red: Nigeria; **D**:wealth tertiles, blue: lowest, red: middle, green highest; **E**: folding net when hanging, blue: never, red: sometimes or always; **F**: cooking in sleeping room, blue: never, red: sometimes or always; **G**: net user: blue: children only, red: child and adult, green: adult only; **H**: gender of head of household: blue: male, red: female.

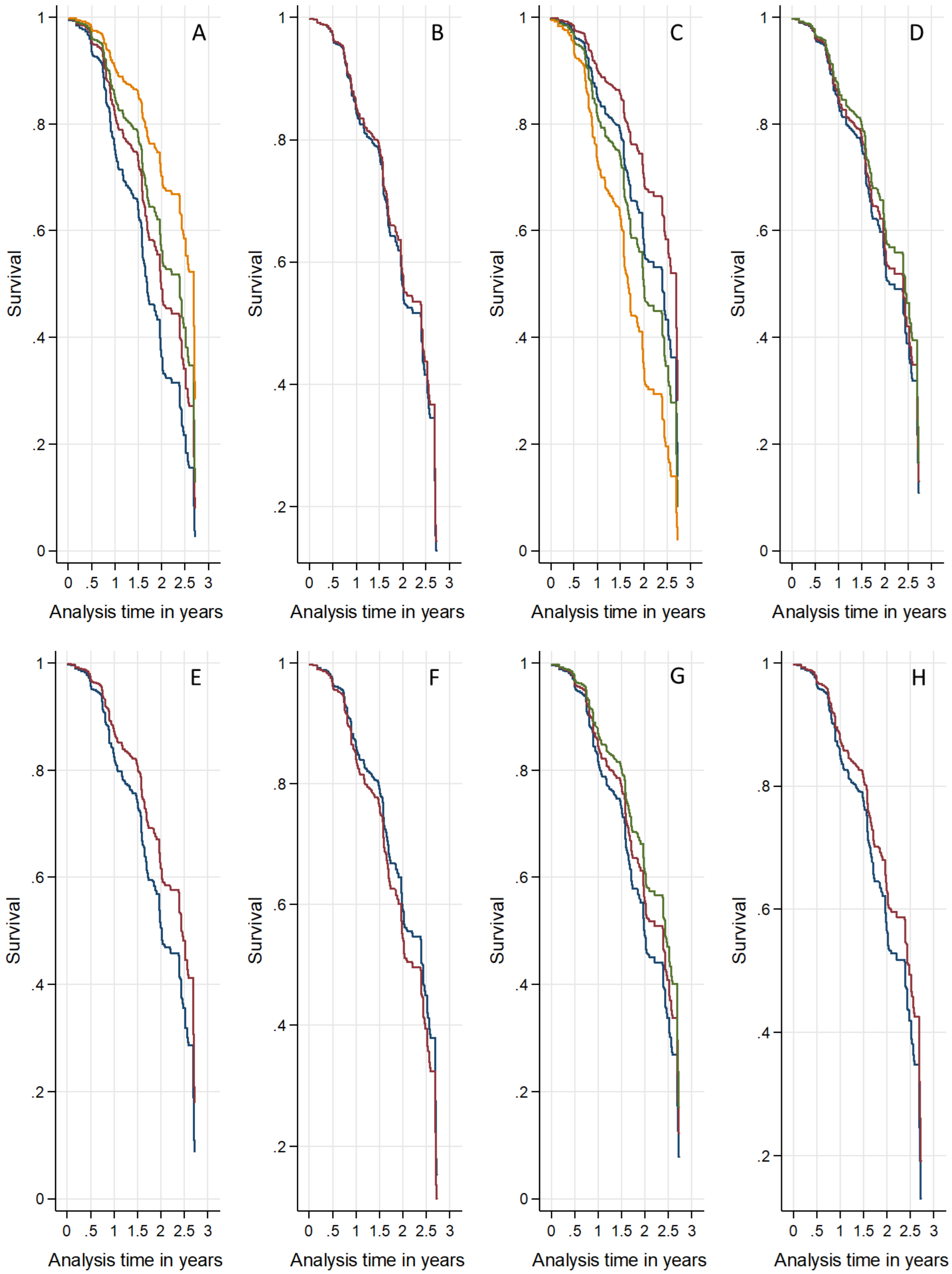

Supplement: Supplementary file 1 — Additional file 1: Figure S1. adjusted survival curves for determinants of physical survival from cox regression. Contains survival graph for each variable in the final Cox regression model. [file 12936_2020_3549_MOESM1_ESM.pdf]
